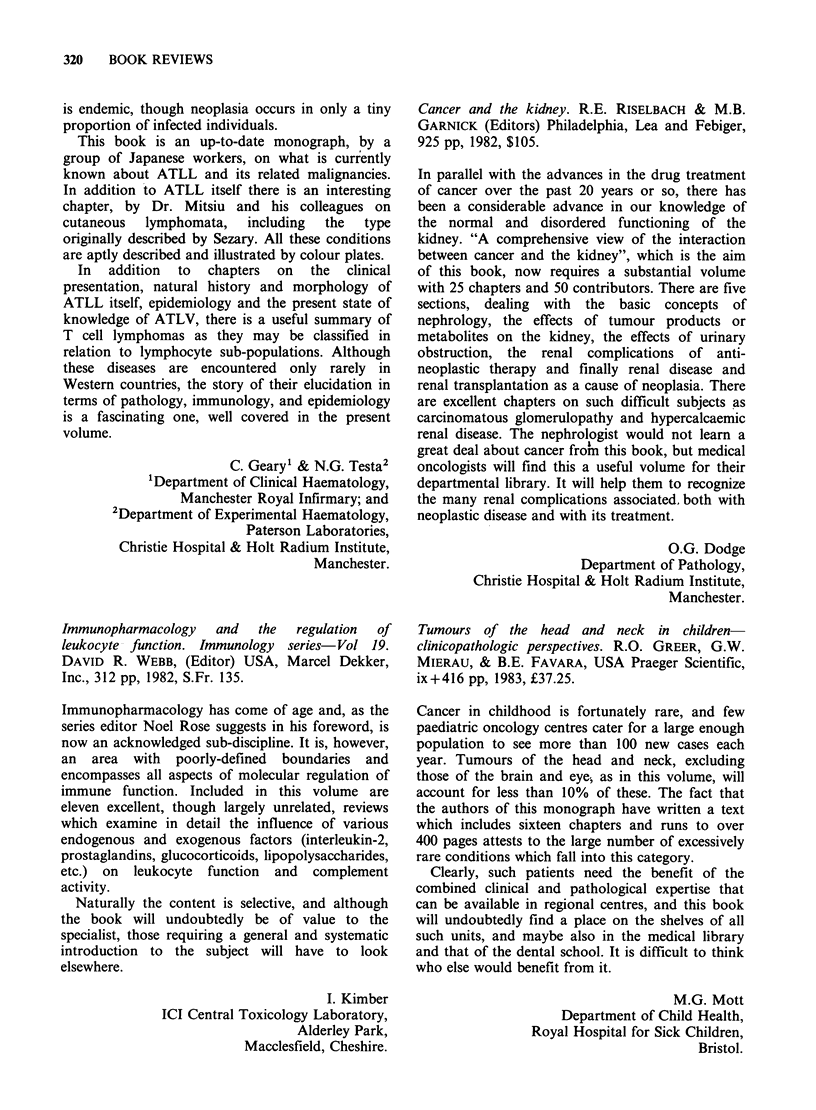# Cancer and the kidney

**Published:** 1983-08

**Authors:** O.G. Dodge


Christie Hospital & Holt Radium Institute,

Manchester.

*Cancer and the kidney.* R.E. Riselbach & M.B. Garnick (Editors) Philadelphia, Lea and Febiger, 925 pp, 1982, $105.

In parallel with the advances in the drug treatment of cancer over the past 20 years or so, there has been a considerable advance in our knowledge of the normal and disordered functioning of the kidney. "A comprehensive view of the interaction between cancer and the kidney", which is the aim of this book, now requires a substantial volume with 25 chapters and 50 contributors. There are five sections, dealing with the basic concepts of nephrology, the effects of tumour products or metabolites on the kidney, the effects of urinary obstruction, the renal complications of anti-neoplastic therapy and finally renal disease and renal transplantation as a cause of neoplasia. There are excellent chapters on such difficult subjects as carcinomatous glomerulopathy and hypercalcaemic renal disease. The nephrologist would not learn a great deal about cancer from this book, but medical oncologists will find this a useful volume for their departmental library. It will help them to recognize the many renal complications associated both with neoplastic disease and with its treatment.

O.G. Dodge
Department of Pathology,
Christie Hospital & Holt Radium Institute,
Manchester.